# The Fanconi Anemia C Protein Binds to and Regulates Stathmin-1 Phosphorylation

**DOI:** 10.1371/journal.pone.0140612

**Published:** 2015-10-14

**Authors:** Audrey Magron, Sabine Elowe, Madeleine Carreau

**Affiliations:** 1 Department of Pediatrics, Université Laval, Québec, QC, Canada; 2 CHU de Québec, CHUL Research Center, Québec, QC, Canada; Institut de Génétique et Développement de Rennes, FRANCE

## Abstract

The Fanconi anemia (FA) proteins are involved in a signaling network that assures the safeguard of chromosomes. To understand the function of FA proteins in cellular division events, we investigated the interaction between Stathmin-1 (STMN1) and the FA group C (FANCC) protein. STMN1 is a ubiquitous cytosolic protein that regulates microtubule dynamics. STMN1 activities are regulated through phosphorylation-dephosphorylation mechanisms that control assembly of the mitotic spindle, and dysregulation of STMN1 phosphorylation is associated with mitotic aberrancies leading to chromosome instability and cancer progression. Using different biochemical approaches, we showed that FANCC interacts and co-localizes with STMN1 at centrosomes during mitosis. We also showed that FANCC is required for STMN1 phosphorylation, as mutations in FANCC reduced serine 16- and 38-phosphorylated forms of STMN1. Phosphorylation of STMN1 at serine 16 is likely an event dependent on a functional FA pathway, as it is reduced in FANCA- and FANCD2-mutant cells. Furthermore, FA-mutant cells exhibited mitotic spindle anomalies such as supernumerary centrosomes and shorter mitotic spindles. These results suggest that FA proteins participate in the regulation of cellular division via the microtubule-associated protein STMN1.

## Introduction

Fanconi anemia (FA) is a rare genetic disorder associated with a progressive failure of the hematopoietic system, generally manifested as anemia, thrombopenia or pancytopenia [1]. In many cases, hematopoietic failure evolves into clonal proliferative diseases such as myelodysplasia or acute myelogenous leukemia [1]. FA patients are also prone to non-hematological malignancies including squamous cell carcinomas [2]. To date, eighteen genes have been associated with FA, and their products are thought to function through a signaling network in response to DNA crosslink damage [3–6]. FA proteins can be divided into three protein complexes that include a multi-protein core complex (FANCA, FANCB, FANCC, FANCE, FANCF, FANCG and FANCL), a two-protein substrate (FANCD2 and FANCI) and downstream effectors (FANCD1, FANCJ, FANCM, FANCN, FANCO, FANCP, FANCQ, FANCS) [7–9]. Mutations in any of these FA and FA-like genes lead to a defective DNA interstrand crosslink (ICL) repair mechanism that result in accumulation of DNA damage. Unrepaired DNA damage interferes with DNA replication and transcription. Replication stress is considered one of the major causes of hematopoietic failure [10, 11]. Other hypotheses put forward to explain bone marrow failure in FA include dysregulated cellular response to inflammatory cytokines, oxidative stress, mitochondrial dysfunction, elevated apoptosis and abnormal cell cycle progression (reviewed in[11]). Dysfunction in any of these mechanisms would negatively impact cellular division of hematopoietic cells.

A number of reports have suggested that FA mutant cells show impaired cellular division characterized by increased cytokinesis failure and defective chromosome segregation [12–16]. Consistent with a role in cellular division, several FA proteins were shown to localize at centrosomes and/or mitotic spindles during mitosis [17–19]. In addition, FANCA was shown to interact with the Never In mitosis A-related kinase (NEK) 2 protein, a kinase involved in maintaining centrosome integrity. FANCA also interacts with the kinetochore-binding domain of the centromere-associated protein (CENP) E [17, 20]. FANCJ, was shown to bind and activate the Polo-Like Kinase-1 (PLK1) to promote centrosome amplification [19]. Furthermore, FANCC was shown to form a complex with the mitotic cyclin-dependent kinase 1 (CDK1), a kinase located at centrosomes and implicated in the initiation of mitosis [21]. Together, these findings suggest that FA proteins participate in the regulation of cellular division acting in centrosome biogenesis. Interestingly, we recently identified the microtubule-associated protein Stathmin-2 (STMN2) and substrate of CDK1, as a putative FANCC-binding partner [22–25].

Stathmin (STMN) is a family of small microtubule-associated proteins involved in cell cycle progression [26, 27]. STMN-1 is the ubiquitous form of the family that includes superior cervical ganglion-10 (SCG10 or STMN2), SCG10-like protein (SCLIP or STMN3), stathmin-like protein B3 (RB3 or STMN4), and two splice variants RB3’ and RB3”, all of which are mostly expressed in the nervous system [28, 29]. All STMN proteins share a highly conserved C-terminus STMN-like domain and a variable N-terminus region. STMN proteins are key regulators of microtubule remodeling due to their direct binding of α/β-tubulin heterodimers, which occurs through the STMN-like domain that acts as a sequestering-tubulin complex [22, 30]. The STMN1-tubulin interaction is regulated through STMN1 phosphorylation on the conserved serine residues namely S16, S25, S38 and S63 [23, 31–33]. This phosphorylation weakens STMN binding to tubulin, as demonstrated by the reduced tubulin affinity of a pseudo-phosphorylated 4-Glu STMN1 mutant [34]. The presence of specific combinations of phosphorylation sites also inhibits STMN1 activity and consequently the binding of tubulin [22, 31]. These findings suggest that unphosphorylated STMN1 binds to and sequesters α/β-tubulin dimers, thus promoting depolymerization of microtubules.

Dysregulation of STMN1 phosphorylation markedly interferes with cellular division as shown by G2/M blockade as well as mitotic spindle disorganization as shown by overexpression of a non-phosphorylable four-serine mutant STMN1 [31, 35, 36]. This inability to assemble a functional mitotic spindle interferes with chromosome segregation, resulting in the formation of micronuclei and cell cycle arrest in the G2/M phase [26, 27, 37]. Therefore, the tight regulation of STMN1 phosphorylation is essential for the process of mitosis. Studies also show elevated levels of STMN1 in many cancers including leukemia, whereas a dramatic decrease in STMN1 levels is found in hematopoietic cells upon differentiation along different lineages [38, 39]. STMN1 has also been implicated in the regulation of polyploidy in megakaryocytes, a function also involving the FA pathway [38, 40]. These findings suggest that STMN1 plays a critical role in cellular division of cell from various tissues including the hematopoietic system and may act in concert with FA proteins.

Here, we show that STMN1 forms a complex and co-localizes with FANCC at centrosomes during mitosis and that FANCC is required for the proper phosphorylation of STMN1 to prevent mitotic spindle abnormalities.

## Materials and Methods

### Plasmids and DNA constructs

The N-terminus of FANCC^1–306^ and the C-terminus of FANCC^307–558^ corresponding to cleaved FANCC fragments were cloned into the pGBKT7 (pGBKFANCC^1–306^ and pGBKFANCC^307–558^) and pGADT7 (pGADFANCC^1–306^ and pGADFANCC^307–558^) yeast vectors as previously described [41, 42]. Yeast pGADFANCC^307-558-L554P^ and pGBKFANCC^307-558-L554P^ constructs were obtained by site-directed mutagenesis using pGADFANCC^307–558^ or pGBKFANCC^307–558^ coding plasmids as templates and the Quikchange II site-directed mutagenesis kit according to the manufacturer’s protocol (Agilent Technologies, Mississauga, ON). The pEGFP-FANCC and pEGFP-FANCC^55–558^ coding for a truncated FANCC mimicking mutations in exon 1 have been previously described [41]. The full-length FANCC harboring the L554P mutation coding plasmid was obtained by site-directed mutagenesis using the pEGFP-FANCC as template (pEGFP-FANCC^L554P^) and the Qickchange II site directed mutagenesis kit. The *STMN1* cDNA was cloned by PCR into pGADT7 and pGBKT7 yeast vectors and subsequently subcloned into pcDNA3.1myc-HisC and the Glutathione-S-transferase fusion pGEX4T1 vectors. STMN1^1–41^ and STMN1^42–149^ coding fragments were cloned by PCR into pGBKT7 and pGADT7 vectors. The STMN1 phospho-mutants S16A, S25A, S38A, and S63A were obtained by site-directed mutagenesis using the Myc-tagged pcDNA3-STMN1 coding plasmid and the Quikchange II site-directed mutagenesis.

### Antibodies

The following antibodies were used: anti-FANCC, (8F3, NovusBiologicals, Littleton, CO), anti-STMN1 (EP1573Y, NovusBiologicals), anti-P16-STMN1 (3353, Cell Signaling Technology, Whitby, ON), anti-P38-STMN1 (ab47399, Abcam, Cambridge, MA or D19H10, Cell Signaling Technology), anti-GFP (clone B2; Santa Cruz Biotechnology, Dallas, TX), anti-α-tubulin (mouse T6199; Sigma-Aldrich), anti-γ-tubulin (mouse T5326, Sigma-Aldrich, Oakville, ON), anti-Myc (9E10, Santa Cruz Biotechnology), anti-Centrin 2 (N-17-R; Santa Cruz Biotechnology), anti-CDK1 (SC-34, Santa Cruz Biotechnology) anti-Aurora-B (NB500-185; NovusBiologicals) and donkey or goat-raised anti-mouse or anti-rabbit Alexa Fluor 488 or 555 (Invitrogen, Burlington, ON).

### Yeast two-hybrid assays

Yeast two-hybrid analyses were performed using the MATCHMAKER Two-Hybrid System 3 according to the manufacturer’s instructions (Clontech, Mountain View, CA) and as previously described [24, 25]. For yeast two-hybrid analyses using FANCC or STMN proteins, yeast constructs were co-transformed into the AH109 *S*. *cerevisiae* strain (Clontech) and selected for growth and reporter gene activation. Positive controls included pGBKT7-p53 with the pGADT7-T antigen. Negative controls were empty pGBKT7 or pGADT7 vectors in combination with the corresponding gene-coding plasmid. Each construct was sequenced and tested for autonomous Gal-4 activation and was found devoid of self-activation. Each experiment was performed at least three times in triplicate with each cDNA cloned into either the pGBKT7 or pGADT7 vector.

### Cells, cultures, transfection and synchronization

HEK293T (CRL-3216, American Type Culture Collection (ATCC), Cerderlane Laboratories Limited, Burlington, ON), HeLa, (CCL-2, ATCC, Cerderlane Laboratories Limited) and FA patient-derived fibroblasts (PD331, PD220 et PD20; Oregon Health and Science University (OHSU) Fanconi Anemia Cell repository with the Fanconi Anemia Research Fund Inc. (FARF), Portland, OR under Dr Markus Grompe’s direction and IRB in accordance with the Department of Health and Human Services regulations at 45CFR46) and the corresponding corrected cells (PD331/C, PD220/A and PD20/D2; OHSU-FA cell repository) were grown at 37°C and 5% CO_2_ in Dulbecco’s modified Eagle’s medium (DMEM) supplemented with 10% fetal calf serum (FCS) as previously described [43]. *FancC* ^-/-^ and wild-type (*FancC* ^+/+^) mouse fibroblast cell lines were obtained from *FancC* knockout mice of mixed C57Bl/6-SV129 background obtained from Dr Manuel Buchwald (Hospital from Sick Children, Toronto, ON, established in accordance with the HSC Ethics review board) as previously described [44]. Cells were transfected using Lipofectamine 2000 (Invitrogen) or the Broad Sprectrum JetPRIME Polyplus Tansfection kit (VWR International, Mississauga ON). For cell synchronization experiments, cells (5×10^5^/mL) were treated either with nocodazol (10 μM for 16 h) or sequentially with 2 mmol/L thymidine for 16 h, thymidine-free media for 9 hours and then 2 mmol/L thymidine for 16 h to arrest cell cycle before analysis.

### Immunoprecipitations and immunoblot analysis

For immunoprecipitation, 5×10^6^ to 1×10^7^ cells were harvested, washed in phosphate-buffered saline (PBS), and resuspended in lysis buffer (50 mM Tris-HCl, 150 mM NaCl, 1% Triton X-100, complete proteases inhibitors [Roche Diagnostics, Laval, QC] and 100 nM okadaic acid). Lysates were cleared by centrifugation and mixed with 1–2 μg of precipitating antibody. Antibody-antigen complexes were pulled down using Dynabeads (Invitrogen) for protein complexes. Immunoprecipitates were resolved by sodium dodecyl sulfate-polyacrylamide gel electrophoresis (SDS-PAGE) on 10%, 12%, or 8–16% gradient polyacrylamide gels, electro-transferred onto a polyvinylidene difluoride (PVDF) membrane and subjected to immunoblotting with specific antibodies, as indicated in each figure. Control immunoprecipitations were performed using either mouse or rabbit IgGs. For immunoblot analysis, total cell lysates were prepared in sodium dodecyl sulfate (SDS)-loading buffer (50 mM Tris-HCl, 2% 2-mercaptoethanol, 2% SDS). Samples were sonicated and/or boiled and subjected to electrophoresis on 10% SDS-polyacrylamide gels. Proteins were electrotransferred onto a PVDF membrane (GE Healthcare Life Sciences, Mississauga, ON) and probed with antibodies.

### Pull-down assays

GST-fusion proteins were prepared following transformation and expression of the GST-tagged protein in *Escherichia Coli*. GST-fusion proteins were immobilized onto sepharose beads and washed before addition of HEK293T cell lysates. Prey-containing lysates were incubated with GST-tagged proteins bound to sepharose beads for 16 h at 4°C. The beads were washed three times (10nM Tris, 150mM NaCl, 1mM EDTA) before elution of protein complexes by boiling samples in loading buffer (125mM Tris, 2.5% β-mercaptoethanol, 2% SDS). Protein complexes were resolved by SDS-PAGE, electrotransferred onto a PVDF membrane, and stained with Ponceau before analysis by immunoblotting procedures as described above.

### Immunofluorescence microscopy

For immunofluorescence microscopy cells were grown in the appropriate culture condition on poly-L-lysine coated coverslips (12-mm diameter). Cells were fixed with 100% methanol for 5 minutes at -20°C, rehydrated overnight at 4°C, and permeabilized for 1 hour with 0.1% saponin and 2% BSA in PBS. Fixed cells were incubated with specific primary antibodies followed by secondary antibodies at the appropriate dilution in PBS with 0.1% saponin and 2% BSA. Following immunofluorescent staining, cells were washed three times with PBS. Slides were mounted with DAPI-fluoromount-G (Southern Biotech, Birmingham, AL) and images were acquired using a Nikon E800 fluorescent microscope equipped with a C1 confocal system (Nikon Canada, Mississauga, ON) or a Carl Zeiss Axio Imager M2 (Zeiss Canada, North York, ON) microscope equipped with an Axiocam MRm (Zeiss) and Axiovision Rel.4.8 software (Zeiss). Images were also acquired using an Olympus IX81-ZDC fluorescent microscope equipped with a Spinning Disc confocal system (Quorum Technologies, Guelph, ON) and Metamorph NX software for 3D image reconstruction. Fluorescence intensity was estimated by pixel number using the ImageJ software (National Institutes of Health).

### Statistical analyses

Data are expressed as mean ± standard errors of the mean (SEM). Statistical analysis was performed using GraphPad Prism software (version 5.0b; GraphPad Software Inc., San Diego, CA). Paired and unpaired two-tailed Student’s t-tests were used to compare groups.

## Results

### STMN interacts with the FANCC protein

**Fig 1:**
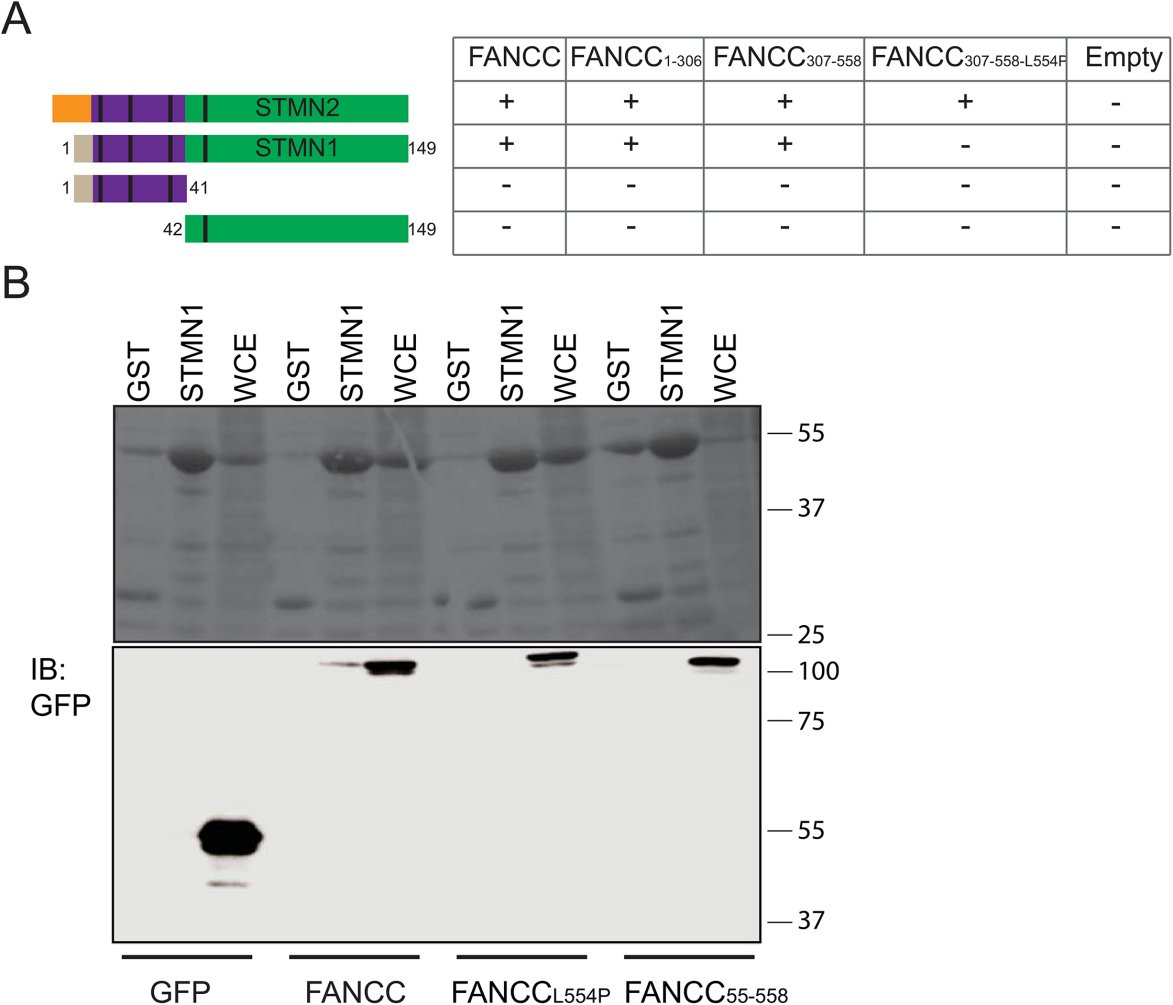
STMN interacts with FANCC. (A) Yeast two-hybrid assays were performed with STMN homologues STMN1 and STMN2 and FANCC constructs. Yeast strain AH109 was co-transformed with full-length STMN2, full-length STNM1 or truncated STMN1 constructs (STMN1^1–41^ and STMN1^42–149^) along with FANCC constructs or empty vectors as indicated. Yeast transforments were assayed for interaction by plating on selective media. Shown are STMN regulatory region (purple) containing the phosphorylation sites (black lines), the interaction domain (green) and subtype-specific N-terminal region of varying size (orange or grey). A positive interaction is indicated as + and a negative interaction with a −. Negative controls included STMN constructs co-transformed with pGADT7 or pGBKT7 empty vectors. Experiments were performed at least 3 times in triplicates. (B) Pull-down experiments using GST-STMN1 (STMN1) or empty-GST (GST) constructs and protein extracts from 293T cells transfected with empty EGFP plasmids or EGFP-FANCC, EGFP-FANCC^L554P^ or EGFP-FANCC^55–558^ expression plasmids. Ponceau stained membrane showing GST or GST-STMN1 fusion proteins (upper panels) and immunoblot (IB) of FANCC constructs using anti-GFP antibodies (lower panels). WCE, whole cell extracts.

In previous studies, we used both N-terminal (FANCC^1–306^) and C-terminal (FANCC^307–558^) regions of FANCC corresponding to cleavage products as baits for yeast two-hybrid analysis and found three independent and strongly positive colonies that encoded for the full-length STMN2 protein [24, 25]. Thus, to further study STMN2 and FANCC interaction, STMN2 was subcloned into pGBKT7 and pGADT7 yeast vectors and retested in yeast two-hybrid assays against FANCC^1–306^, FANCC^307–558^, and full-length FANCC. We found that STMN2 interacts with both N- and C-terminal regions of FANCC as well as full-length FANCC in yeasts (Fig 1A), suggesting that various protein domains of FANCC mediate STMN2 binding.

Because our yeast two-hybrid screens were performed with a fetal brain cDNA library [24, 25], we identified STMN2, the neuron-specific homologue of the STMN family of proteins. To determine whether FANCC interacts with the ubiquitous STMN1, we performed yeast two-hybrid assays using STMN1 as bait with FANCC^1–306^, FANCC^307–558^, and full-length FANCC. Similar to STMN2, we found that STMN1 interacts with FANCC^1–306^, FANCC^307–558^, and full-length FANCC (Fig 1A). These findings indicate that FANCC N- and C-terminal domains effectuate the interaction with both STMN family members, STMN1 and STMN2.

Next, to determine whether specific STMN1 domains mediate its interaction with FANCC, STMN1 functional domains were cloned into yeast vectors, including the regulatory domain containing three of the four characterized phosphorylation sites, the STMN-like domain and the Proline rich domain (STMN1^1–41^) and the C-terminal region (STMN1^42–149^) containing the two tubulin-binding domains [45]. These domains were tested in yeast two-hybrid assays against FANCC^1–306^, FANCC^307–558^, and full-length FANCC. Surprisingly, no positive interactions were found between STMN1^1–41^ or STMN1^42–149^ and FANCC constructs, as shown by negative growth on stringent nutritional selection (Fig 1A) suggesting that FANCC-STMN1 interaction in yeasts requires full-length STMN1.

To determine whether a FA-causing mutation of FANCC impacts its ability to interact with STMN1, we generated a FANCC C-terminus construct harboring the L554P mutation (FANCC^307-558-L554P^) found in patients with FA. We found that the interaction between STMN2 and FANCC^307-558-L554P^ still occurred in yeasts (Fig 1A), but no interactions were found between FANCC^307-558-L554P^ and STMN1, STMN1^1–41^, or STMN1^42–149^ (Fig 1A), suggesting that this mutated form of FANCC maintains its binding ability with the neuron-specific STMN2 but loses its ability to interact with the ubiquitous STMN1.

To biochemically confirm the fidelity of the STMN1-FANCC interaction, we performed GST-mediated pull-down experiments. We generated glutathione (GST)-STMN1 fusion proteins that were immobilized on sepharose beads and incubated with cellular protein extracts prepared from HEK293T cells overexpressing green fluorescence protein (GFP)-tagged full length FANCC or mutated forms of FANCC (FANCC^L554P^ and FANCC^55–558^). The FANCC^55–558^ construct mimics the 50-kDa amino acid terminal-truncated FANCC polypeptide resulting from translation restart at methionine 55, which occurs in cells from patients with a 322delG mutation [46]. Cell lysates contained the expected GST-tagged STMN1 proteins or controls (Fig 1B, upper panel). Western blot analysis using anti-GFP antibodies revealed an interaction between full-length wild-type FANCC and STMN1, as shown by the presence of GFP-FANCC proteins in STMN1-mediated pulled-down protein complexes, whereas no GFP controls were pulled down with STMN1 (Fig 1B, lower panel). No interactions were detected between STMN1 and mutant forms of FANCC notably, FANCC^L554P^ and FANCC^55–558^. These results indicate that FANCC interacts with STMN1 and that disease-causing mutations in FANCC may prevent this interaction.

### FANCC co-localizes with STMN1 at centrosomes

**Fig 2:**
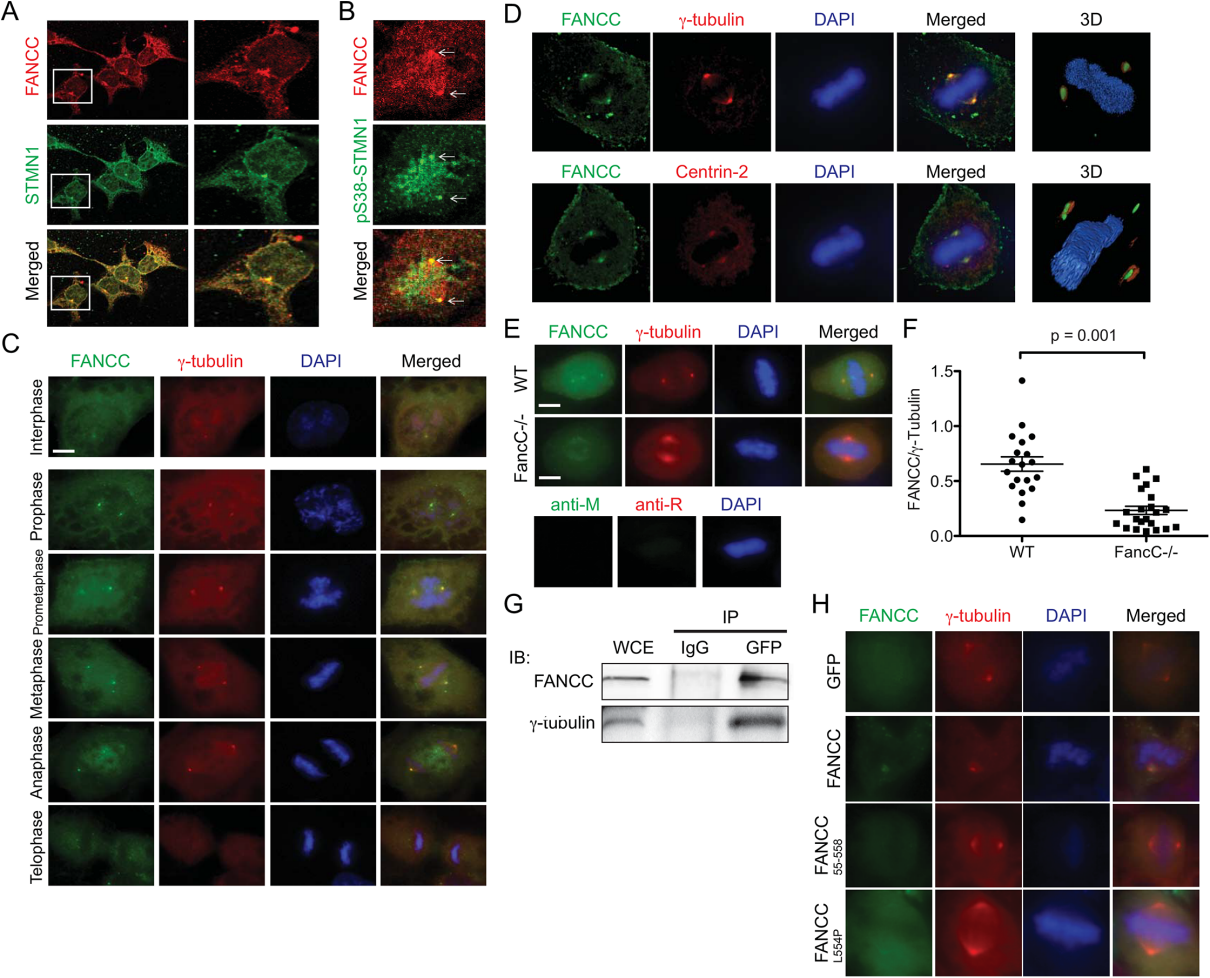
FANCC co-localizes with STMN1 at centrosomes. (A) HeLa cells double-stained with anti-FANCC (red) and anti-STMN1 (green) antibodies were visualized by confocal microscopy using a Nikon E800 microscope equipped with a C1 confocal system at 100x magnification. Data shown are representative of 3 experiments in which at least 20 cells were analyzed. The white squares indicate the zoomed area shown on the right. (B) Immunofluorescence microscopy of mitotic HeLa cells labeled with anti-FANCC and anti-phospho-serine 38 STMN1. Arrows indicate centrosomes. (C) Immunofluorescence microscopy of HeLa cells during the different phases of mitosis as indicated. Cells were labeled with anti-FANCC (green) and anti-γ-tubulin (red) antibodies. (D) Confocal microscopic visualization and 3D reconstruction of 293T cells labeled with anti-FANCC and anti-γ-tubulin (upper panel) or anti-Centrin-2 (lower panel) antibodies using a Olympus IX81-ZDC fluorescent microscope equipped with a Spinning Disc confocal system and the Metamorph NX software. (E) Wild type (WT) and *FancC* ^-/-^ fibroblasts labeled with anti-FANCC (green) and anti-γ-tubulin (red) antibodies (upper panel). HeLa cells labeled with control IgG followed by secondary antibodies as indicated (Lower panel). (F) FANCC fluorescence intensity relative to γ-tubulin at centrosomes scored in at least 20 mitotic cells from each genotype. P value is indicated in the graph. (G) Immunoprecipitation of EGFP-FANCC with endogenous γ-tubulin from EGFP-FANCC transfected 293T cells and immunoprecipitated with anti-GFP antibodies or control IgG (mouse). Immunoprecipitates were immunoblotted with anti-γ-tubulin or anti-FANCC antibodies. Shown is a representative experiment out of 3. (H) 293T cells were transfected with EGFP-FANCC constructs as indicated in the figure prior to immunofluorescence detection. Cells were labeled with anti-γ-tubulin (red) antibodies. Cells were visualized with a Carl Zeiss Axio Imager M2 at 100X magnification. Nuclear DNA was stained with DAPI. White line indicates scale of 1 μm.

STMN1 is located in the cytoplasm during interphase and becomes concentrated at the mitotic spindle during cellular division [47]. Therefore, we sought to determine whether the localization of FANCC changes concomitantly with STMN1 across the cell cycle. First, immunofluorescence staining and confocal microscopy analysis of FANCC and STMN1 in cells were performed. Results showed that FANCC and STMN1 are localized in the cytoplasm during interphase (Fig 2A). In view of the fact that STMN1 becomes phosphorylated in mitotic cells, we performed immunostaining experiments using a phospho-specific anti-STMN1 antibody. As expected, results showed localization of the phosphorylated form of STMN1 (pS38-STMN1) at centrosomes (Fig 2B). Surprisingly, we observed strong labeling of FANCC at centrosomes (Fig 2B). To confirm that FANCC localizes at centrosomes, we performed co-immunostaining of FANCC along with a centrosomic protein, γ-tubulin, in cells at different cell cycle phases. We observed strong immunostaining of FANCC with γ-tubulin at centrosomes in cells from prometaphase to anaphase (Fig 2C). We next performed 3D reconstructions of 2D confocal image stacks of mitotic cells and showed that FANCC co-localized with γ-tubulin at centrosomes (Fig 2D, upper panels; S1 Video) and also co-localized with the centrosome-specific protein Centrin-2 (Fig 2D, lower panels; S2 Video). These results provide additional evidence that FANCC localizes to centrosomes during mitosis. The lack of immunostaining in the centrosomes of *FancC* ^-/-^ knock-out cells with anti-FANCC antibodies or secondary antibodies alone (Fig 2E and 2F) demonstrates that the localization of FANCC to centrosomes is not a result of non-specific antibody staining. In addition, co-immunoprecipitation experiments showed that GFP-FANCC forms a complex with endogenous γ-tubulin (Fig 2G). Together, our results suggest that FANCC forms a complex with STMN1 at centrosomes during mitosis.

Because mutant forms of FANCC fail to interact with STMN1, we wished to determine whether mutations in FANCC also affected the centrosomic location during mitosis. We thus performed immunofluorescence experiments using GFP-tagged FANCC mutant constructs. Microscopic analysis of FANCC constructs showed that mutant forms of FANCC, GFP-FANCC^L554P^ and GFP-FANCC^55–558^, fail to localize at centrosomes (Fig 2H). These results suggest that mutations in FANCC prevent its interaction and localization with STMN1 at centrosomes.

### STMN1 phosphorylation requires FANCC

**Fig 3:**
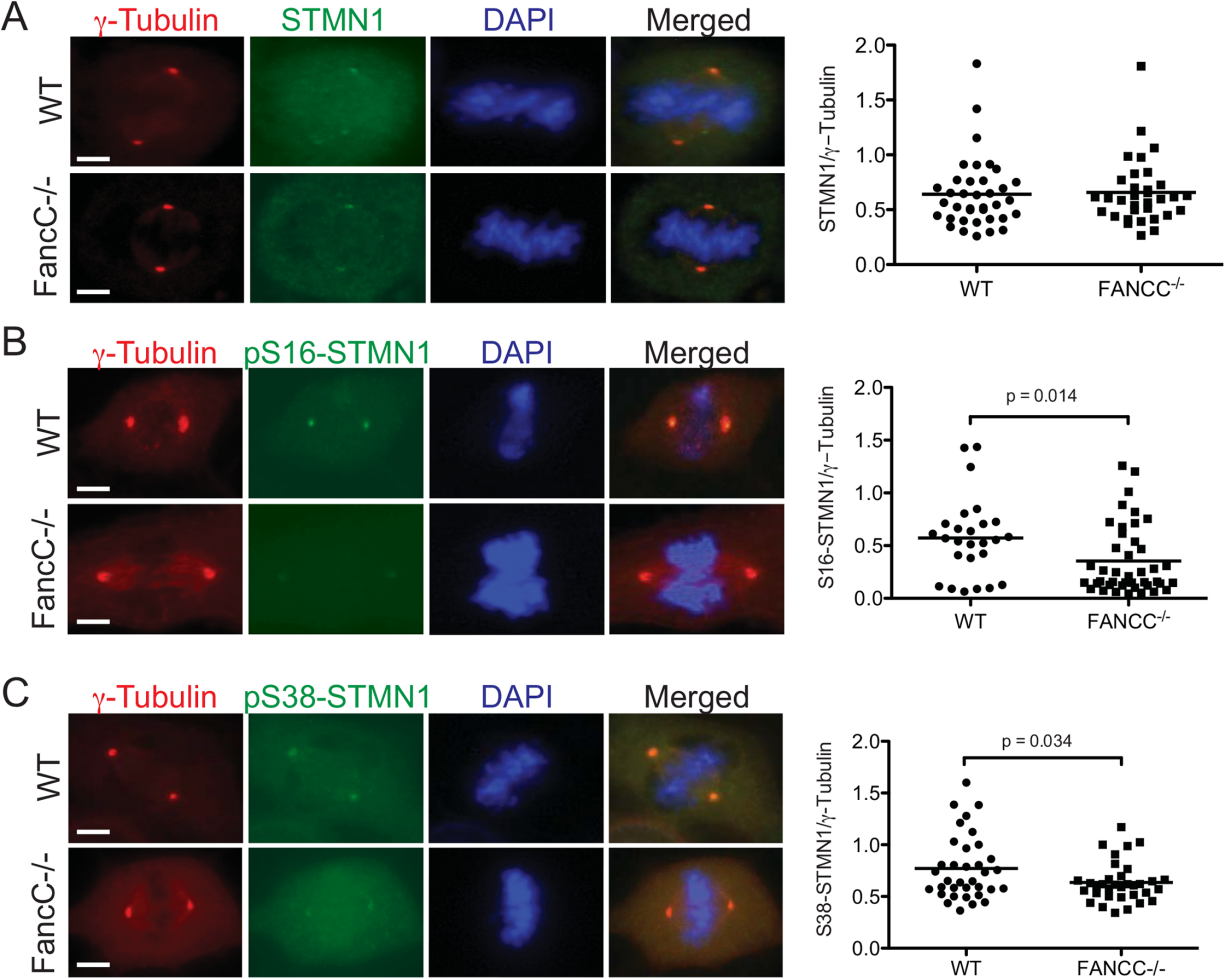
STMN1 phosphorylation requires FANCC. (A-C) *FancC* ^-/-^ and wild-type cells were immunostained with antibodies against total STMN1 (in A), STMN1-phospho-Serine 16 (in B), or STMN1-phospho-serine 38 (in C) along with γ-tubulin during mitosis and visualized using a Carl Zeiss Axio Imager M2 at 100X magnification. White line indicates scale of 1 μm. Ratio of pS16-STMN1, pS38-STMN1 or STMN1 fluorescence intensity relative to γ-tubulin at centrosomes were scored in at least 20 mitotic cells each from 3 separate experiments. P values are indicated in each graph.

Regulation of STMN1 phosphorylation is essential for the process of mitosis and in view of FANCC interaction with STMN1 and cell cycle defects in FA cells, we examined whether FANCC is required for STMN1 phosphorylation during mitosis. Cells obtained from *FancC* ^-/-^ knock-out mice and wild-type littermates were cultured and arrested in mitosis. Cells were then fixed and co-labeled with anti-STMN1 or anti-phospho-STMN1 antibodies together with anti-γ-tubulin antibodies and visualized using fluorescence microscopy. STMN1 phosphorylation levels at centrosomes were evaluated by measuring STMN1 fluorescence intensity relative to γ-tubulin fluorescence intensity using the Axiovision acquisition software. Centrosomes from at least 20 mitotic cells were analyzed for each condition. *FancC* ^-/-^ and wild-type cells showed similar total STMN1 protein levels at centrosomes (Fig 3A). However, both S16- and S38-phosphorylated forms of STMN1 were significantly reduced at centrosomes of *FancC* ^-/-^ cells compared with wild-type cells (Fig 3B and 3C), suggesting that FANCC is required for efficient STMN1 phosphorylation during mitosis.

**Fig 4:**
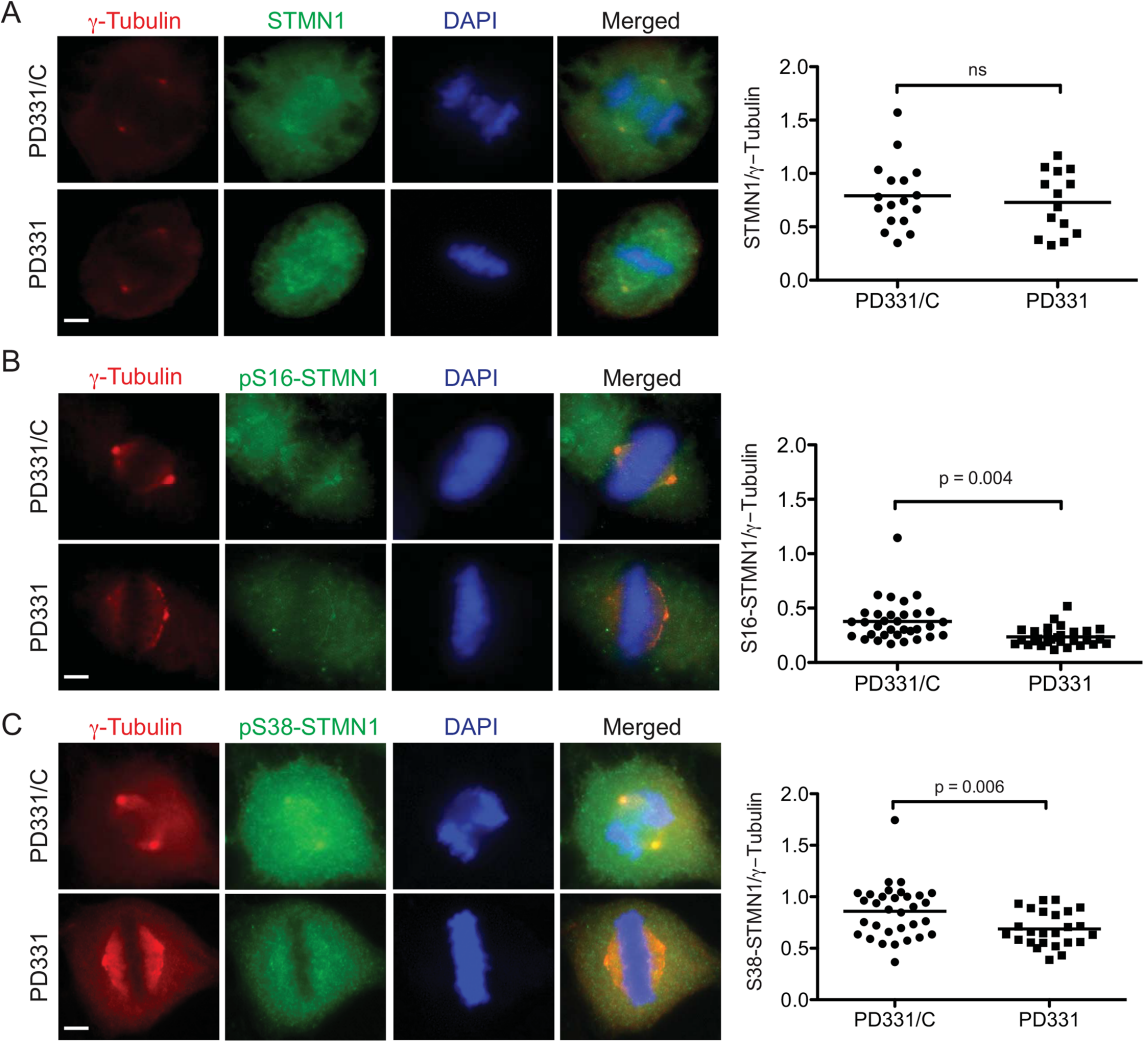
STMN1 phosphorylation requires FANCC. FANCC-mutant cells PD331 and FANCC-corrected cells (PD331/C) were immunostained with antibodies against total STMN1 (A), STMN1-phospho-Serine 16 (in B), or STMN1-phospho-serine 38 (in C) along with γ-tubulin during mitosis and visualized using a Carl Zeiss Axio Imager M2 at 100X magnification. White line indicates scale of 1 μm. Ratio of total STMN1, pS16-STMN1 or pS38-STMN1 fluorescence intensity relative to γ-tubulin at centrosomes were scored in at least 20 mitotic cells each from 3 separate experiments. P values are indicated in each graph.

**Fig 5:**
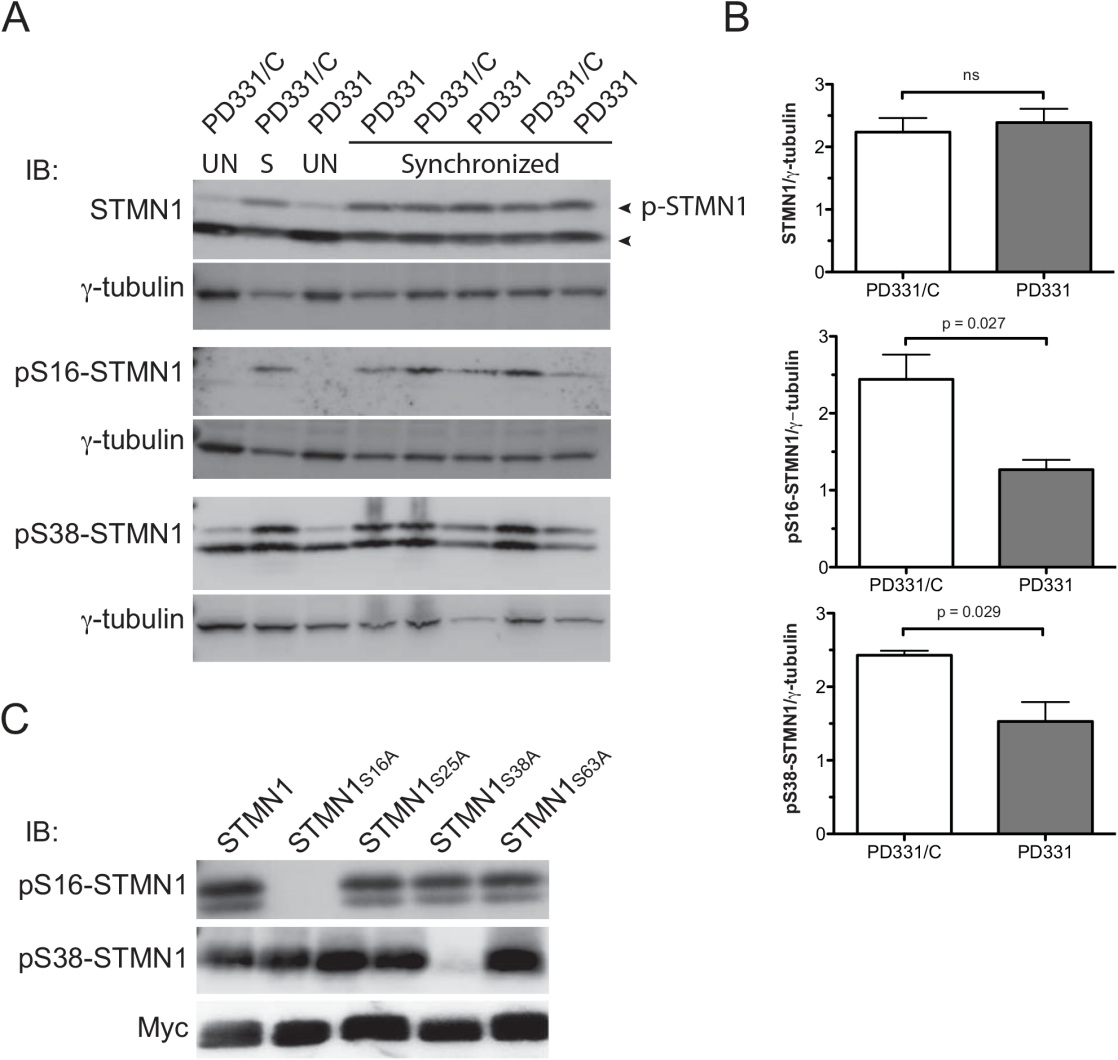
Reduced levels of phosphorylated STMN1 in FANCC mutant cells. (A) Western blot analysis of protein extracts from FANCC mutant (PD331) and FANCC-corrected (PD331/C) cells. Cells were unsynchronized (UN) or synchronized (S) using nocodazol (10 μM for 16 hours). Total protein extracts were immunoblotted (IB) with the indicated antibodies. Arrows indicate total STMN1 and STMN1 phosphorylated species. (B) Graph displaying the mean relative ratio of STMN1/γ-tubulin protein expression from 3 separate experiments. (C) Western blot analysis of protein extracts from cells overexpressing serine-to-alanine Myc-tagged STMN1 mutants. Total protein extracts were immunoblotted (IB) with the indicated antibodies

To determine whether FA-causing mutations affect STMN1 phosphorylation status, we performed similar experiments using patient-derived FANCC mutant cells (PD331) and the corrected cells (PD331/C). Like murine *FancC* ^-/-^ knock-out cells, PD331 and PD331/C cells showed similar total STMN1 levels at centrosomes (Fig 4A), whereas a significant decrease in S16- and S38-phosphorylated forms of STMN1 was observed in PD331 cells compared with PD331/C cells (Fig 4B and 4C). These results suggest that mutation in or absence of FANCC reduces phosphorylated forms of STMN1 at centrosomes during mitosis. To confirm these results, we performed western blot analysis of protein extracts from mitotic PD331 and PD331/C cells. Cells were synchronized with nocodazole prior to protein extraction and immunoblotting to induce STMN1 phosphorylation. PD331 and PD331/C mitotic cells showed similar total STMN1 protein levels, whereas S16- and S38-phosphorylated forms of STMN1 were reduced in PD331 compared with PD331/C cells (Fig 5A and 5B). To ensure the specificity of the anti-phospho-STMN1 antibodies, we generated STMN1 serine-to-alanine mutants of S16, S25, S38, and S63. Western blot analysis of protein extracts from cells overexpressing these mutants confirmed the specificity of the antibodies (Fig 5C). Together, these results suggest that FANCC is required for efficient STMN1 phosphorylation, at least at the S16 and S38 residues.

### A functional FA pathway is required for efficient STMN1 phosphorylation

**Fig 6:**
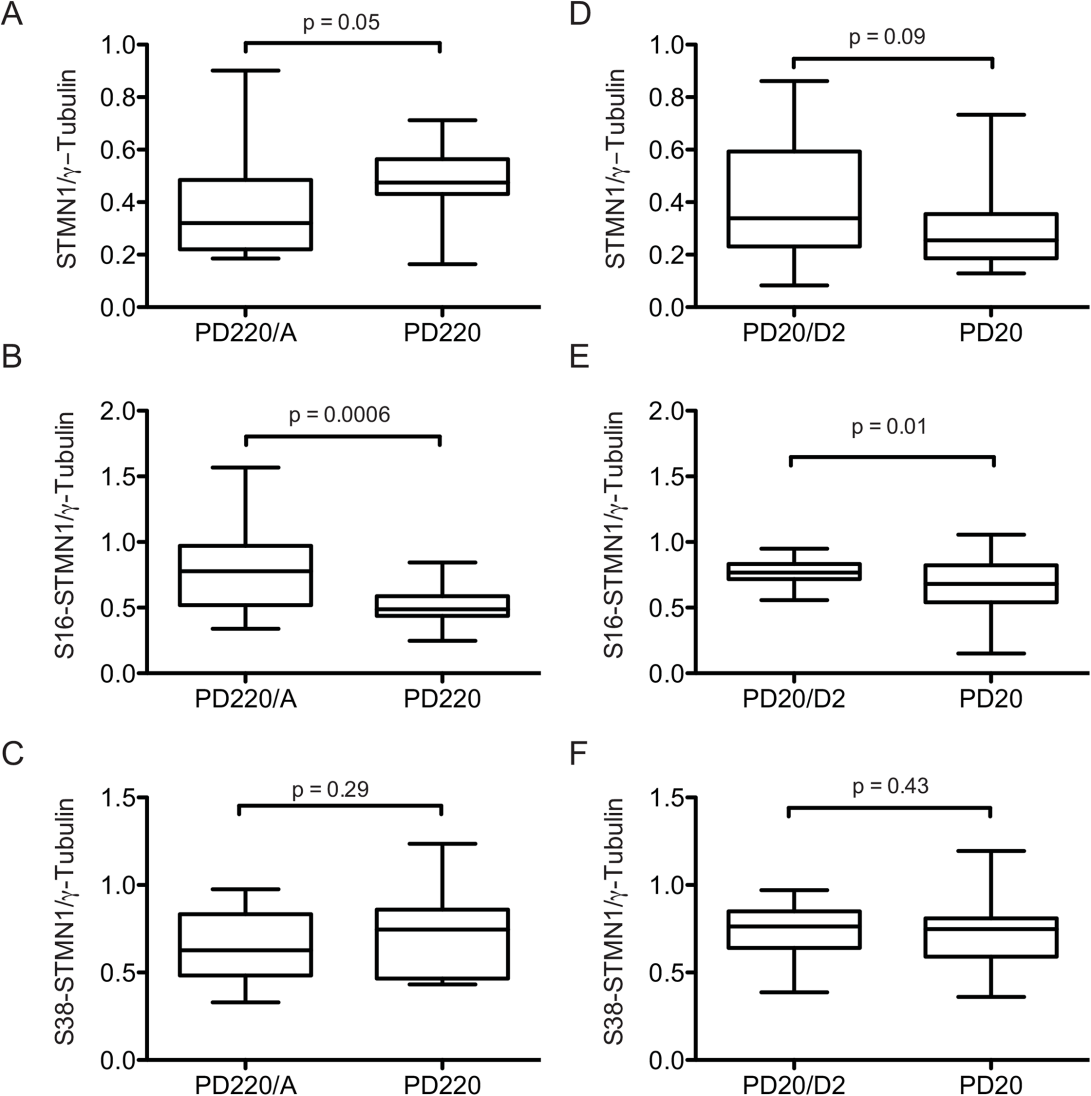
STMN1 phosphorylation depends on a functional FA pathway. Graphs displaying the ratio of total STMN1, pS16-STMN1 or pS38-STMN1 fluorescence intensity relative to γ-tubulin at centrosomes in FA patient-derived PD220 and PD20 cells ant their corrected counterparts PD220/A and PD20/D2 cells. Intensity of at least 20 mitotic cells were scored for each cell line from 3 separate experiments. P values are indicated in each graph.

To determine whether STMN1 phosphorylation during mitosis is uniquely dependent on FANCC or also dependent on the FA pathway (other FA proteins), we evaluated S16- and S38-phosphorylated forms of STMN1 in patient-derived FANCA and FANCD2 mutant cells (PD220 and PD20, respectively). We chose FA group A to represent another core-complex protein and group D2 as a downstream FA pathway component. We performed experiments where PD220 and PD20 cells were synchronized by thymidine blockage, fixed, co-labeled with anti-STMN1 or anti-phospho-specific STMN1 antibodies along with anti-γ-tubulin antibodies. Labeled cells we visualized using fluorescence microscopy and fluorescence intensity ratio of STMN1 to γ-tubulin were estimated. The FANCA-mutant cells PD220 showed significantly higher levels of STMN1 at centrosomes compared to the FANCA-corrected cells, whereas FANCD2-mutant cells (PD20) showed similar total STMN1 levels at centrosomes compared with the FANCD2-corrected counterparts (Fig 6A and 6D and S1 Fig). However, a significant decrease in S16-phosphorylated forms of STMN1 was observed in both PD220 and PD20 cells compared to the corrected counterparts (Fig 6B and 6E and S1 Fig). No significantly difference in S38-phosphorylated forms of STMN1 were observed between mutant and corrected FA cells (Fig 6C and 6F and S1 Fig). These results suggest that STMN1 phosphorylation requires a functional FA pathway for at least the S16 residue whereas S38-phosphorylation necessitates FANCC.

**Fig 7:**
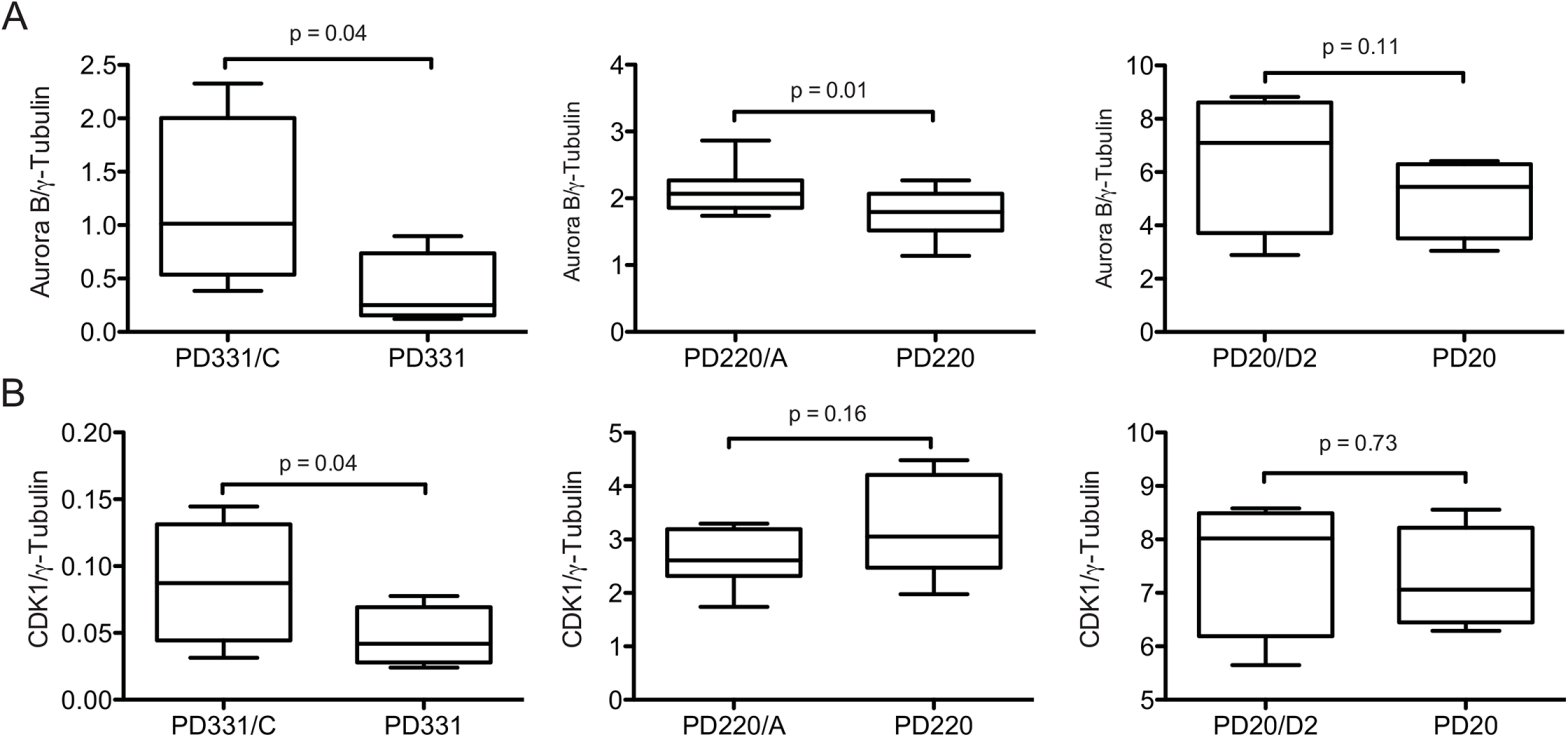
Reduced kinases expression in FA-deficient cells. (A-B) Graph displaying the mean relative ratio of Aurora B/γ-tubulin (in A) and CDK1/γ-tubulin (in B) protein expression from 3 separate experiments each from PD331, PD331/C, PD220, PD220/A, PD20 and PD20/D2 cells. P values are indicated in the graphs.

STMN1 is a target of many kinases [22, 48, 49] and in view of its reduced phosphorylation in FA cells, performed Western blot analysis of the two kinases Aurora B and CDK1 involved in S16- and S38-STMN1 phosphorylation respectively. Results show that Aurora B expression was significantly reduced in PD331 and PD220 cells compared to the corresponding corrected cells, whereas a slight reduction in protein expression was observed in PD20 compared to PD20/D2 cells (Fig 7A). These results indicate that Aurora B involved in STMN1 S16-phosphorylation is reduced in FA-deficient cells and correlate with reduced phospho-S16-STMN1 observed in FA-deficient cells. In addition, we observed reduced CDK1 protein levels in PD331 cells compared to the corrected PD331/C cells, whereas no differences were observed between PD220 or PD20 cells and their corrected counterparts (Fig 7B). These results correlate with reduced S38-phospho-STMN1 found only in FANCC-deficient cells.

### Spindle abnormalities in FA-deficient cells

**Fig 8:**
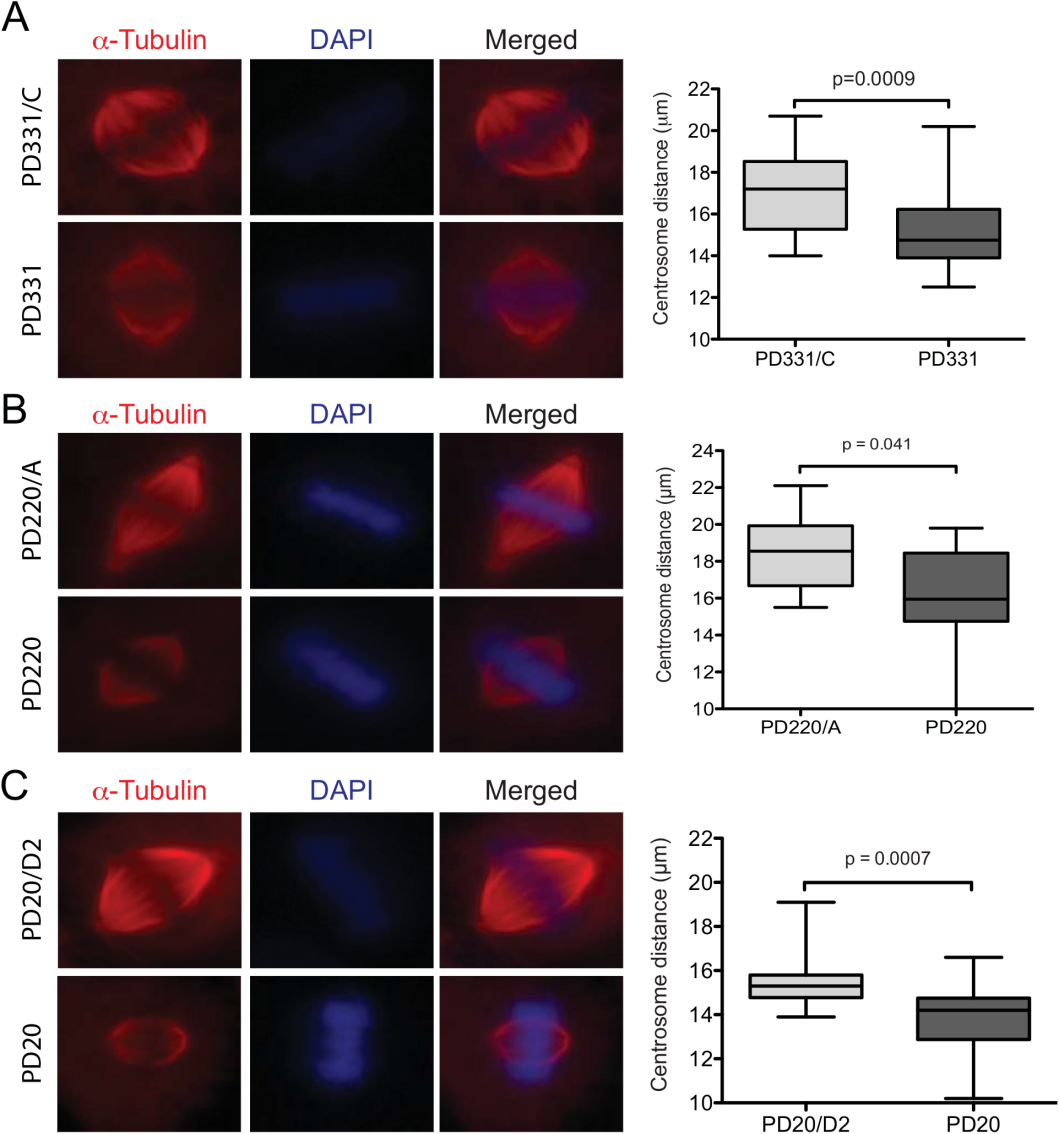
Reduced mitotic spindle size in FA-deficient cells. Immunofluorescence staining of α-tubulin in synchronized PD331 and FANCC-corrected PD331/C cells (A), PD220 and FANCA-corrected PD220 cells (B) and PD20 and FANCD2-corrected PD20 cells (C). Nuclear DNA was labeled with DAPI. Cells were visualized using a Carl Zeiss Axio Imager M2 at 100X magnification. Distance between centrosomes was evaluated in at least 22 mitotic cells from 3 separate experiments using the Axiovision Rel.4.8 software (Zeiss). P values are indicated in the graphs.

**Fig 9:**
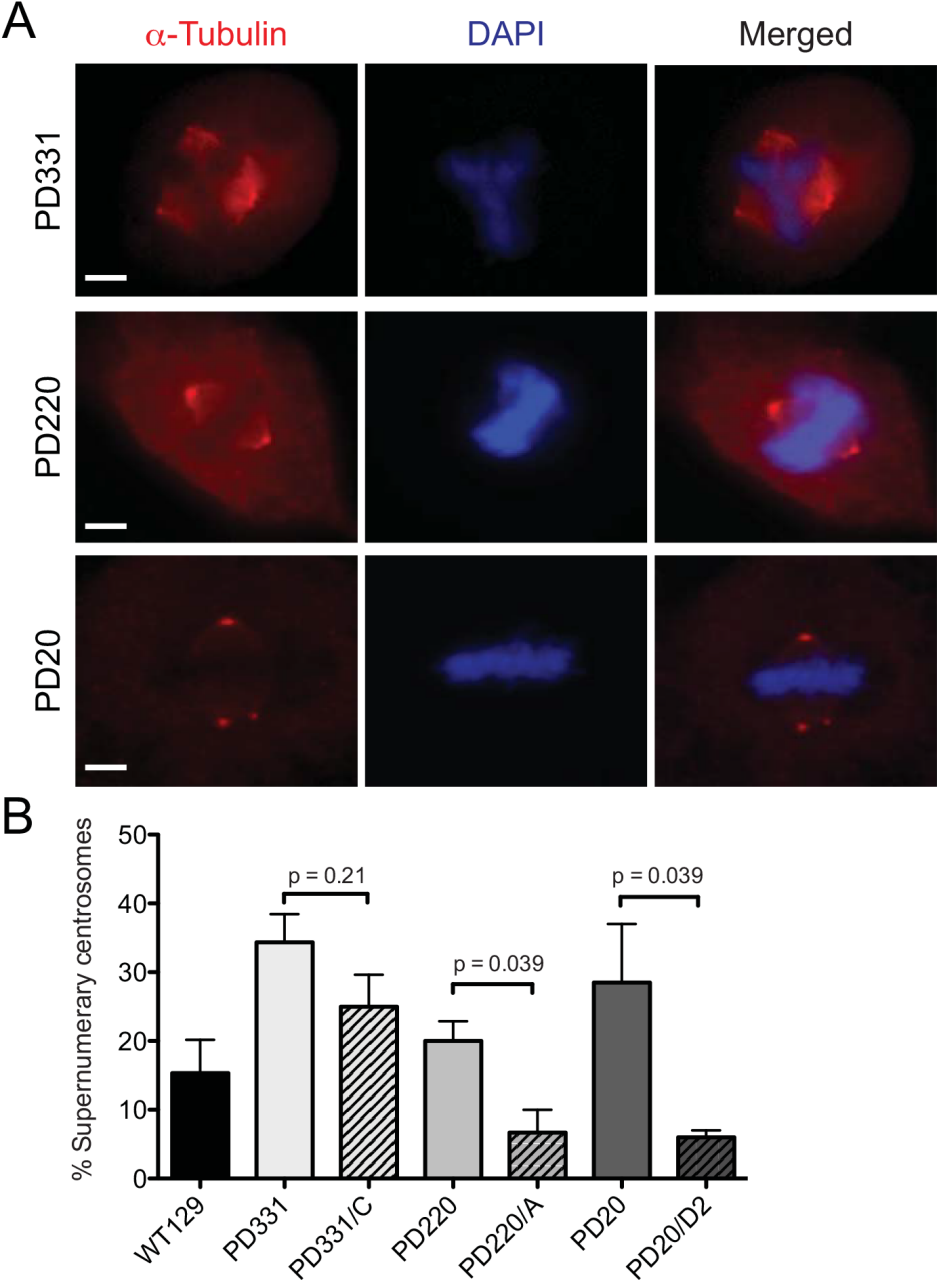
Supernumerary centrosomes in FA-deficient cells. Representative immunofluorescence staining of α-tubulin in FA-C (PD331), FA-A (PD220) and FA-D2 (PD20) mitotic cells. Nuclear DNA was labeled with DAPI. (B) Percent number of cells with supernumerary centrosomes measured in at least 50 mitotic cells of each normal WT129 cells and patient-derived PD331, PD220 and PD20 cells and their corrected counterparts from 3 separate experiments. P values are indicated in the graph.

Altered STMN1 phosphorylation is associated with mitotic spindle abnormalities, including reduced spindle size and stability [36]. Because FA proteins appear to be required for efficient STMN1 phosphorylation during mitosis, we first evaluated mitotic spindle sizes in FA-deficient cells. FA-deficient cells were synchronized, fixed, labeled with anti-α-tubulin antibodies and evaluated by fluorescence microscopy. The distance between centrosomes in cells was evaluated by calculating pixel sizes between centrosomes. As expected, all FA patient-derived cells tested showed significantly shorter spindle sizes during mitosis compared to their respective corrected cells (Fig 8). Next, we evaluated the number of cells containing more than two centrosomes during mitosis. Results show that FA-mutant cells had elevated numbers of cells with supernumerary centrosomes compared to normal control WT129 cells, where 35% of PD331 and 25% of PD20 cells showed more than two centrosomes (Fig 9). Although PD220 cells showed elevated numbers of cells with more than two centrosomes, the difference between WT129 and PD220 cells was not significant (Fig 9B). Our results suggest that the absence of or mutation in FA proteins, which affect STMN1 phosphorylation lead to mitotic spindle abnormalities.

## Discussion

In this study, we found that the Fanconi anemia protein, FANCC interacts directly with the microtubule-associated protein STMN1, involved in microtubule dynamics during cellular division. Previous studies have hinted at a role of FA proteins in the regulation of cellular division. For instance, FA patient-derived cells and cells from knock-out mouse models of FA show abnormal chromosome segregation, cytokinesis failure and perturbed cell cycle progression through mitosis [13, 14, 50, 51]. The present findings provide further evidence supporting the role of FA proteins in cellular division by showing that FANCC co-localizes with STMN1 at centrosomes and that mutations in FANCC prevent this localization. Previous studies have also showed the presence of FA proteins at centrosomes and/or the mitotic spindle. For instance, one study showed the presence of FANCC at mitotic spindles [18]. Although we observed FANCC at mitotic spindles, we found that the FANCC signal was stronger at centrosomes. Our results differ from those of Nalepa *et al* possibly because of the different methods of fixation and/or antibodies used for FANCC labeling. Nevertheless, these studies combined with our results suggest that FANCC acting with STMN1 is critical for centrosome function. The fact that STMN1 regulates mitotic division through the regulation of microtubule assembly and disassembly suggests that failed interaction between FANCC and STMN1 in FA patient-derived cells would result in mitotic defects. Indeed, we showed that FA-deficient cells present shorter spindle sizes and supernumerary centrosomes. Previous studies have showed that disruption in FA signaling is associated with abnormal centrosome numbers [17, 18, 52]. Consistent with a role in centrosome maintenance, FA proteins were shown to interact with regulators of centrosome biogenesis, these include NEK2, CENPE and Polo-Like Kinase-1 (PLK1) [17, 19, 20]. Furthermore, FANCC was shown to form a complex with the mitotic cyclin-dependent kinase 1 (CDK1), a kinase located at centrosomes and implicated in the initiation of mitosis [21]. Together, these findings support the notion that FA proteins participate in the regulation of cellular division.

STMN1 has been shown to regulate microtubule formation at centrosomes thus regulating the onset of mitotic spindle assembly and disassembly required for mitotic progression [53, 54]. To this end, STMN1 activity is tightly regulated by phosphorylation during mitosis [55]. While STMN1 is found in a non-phosphorylated state at interphase, it becomes hyperphosphorylated on four serine residues in mitotic cells. Several studies attempting to identify the stoichiometry of phosphorylated forms of STMN1 have shown a sequential phosphorylation of STMN1 on S25/S38 as a prerequisite for S16/S63 phosphorylation. [23, 31, 32, 36, 56]. These studies are supported by structural analysis showing that, when bound to tubulin dimers, STMN1 serine residues 25 and 38 are more accessible and phosphorylation of these residues facilitate access to Ser16 and Ser63 [57]. S63-phosphorylation causes strong structural changes in STMN1, which prevent its tubulin-binding activity thus favoring mitosis [33]. In our study, we observed reduced STMN1-S38-phosphorylation in FANCC but not FANCA and FANCD2-deficient cells suggesting that this phosphorylation event is dependent on a functional FANCC. However, we found that S16-mediated phosphorylation of STMN1 was reduced in all FA patient-derived cells tested suggesting that efficient STMN phosphorylation requires a functional FA signaling pathway. Altered STMN1 phosphorylation is associated with mitotic spindle abnormalities and changes in M phase progression, leading to an accumulation of cells at the G2/M phase [23, 49]. In addition, mutant forms of STMN1 that prevent phosphorylation are associated with shorter mitotic spindles and perturbed spindle morphology [36, 55]. These cellular features of a deregulated STMN1 activity are reminiscent of those found in FA patient’s cells. Thus, it is tempting to speculate that a possible role of FA proteins may be to favor phosphorylation of STMN1 and allow proper mitotic spindle formation.

**Fig 10:**
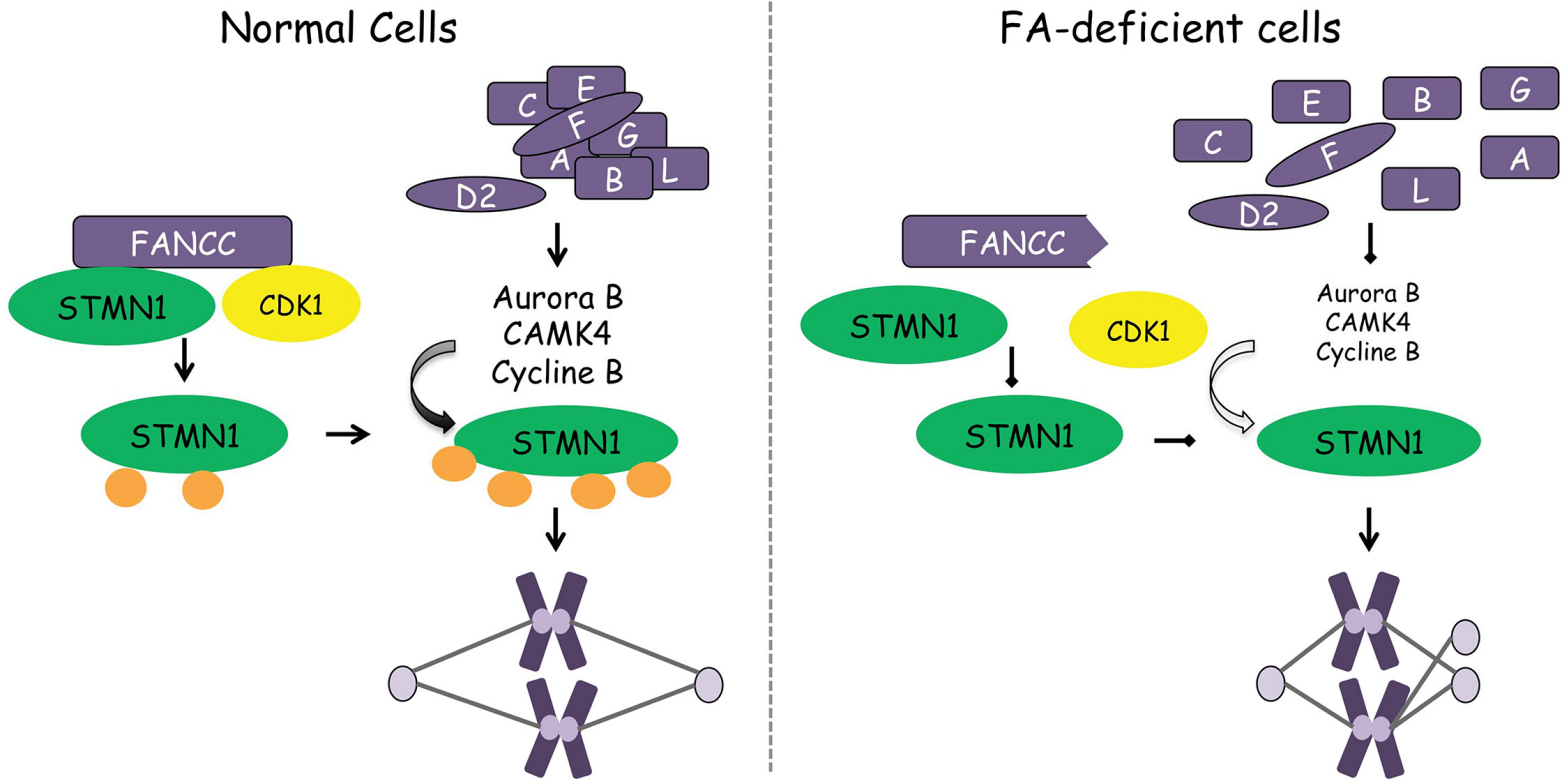
Schematic representation of FA-mediated regulation of STMN1. In normal cells (left panel), FANCC interacts with STMN1 and facilitates the action of CDK1 for STMN1 phosphorylation on serine 38. Subsequent phosphorylation of STMN1 requires a functional FA signaling pathway to ensure proper centrosome number and mitotic spindle size. In FA-deficient cells (right panel), lack of interaction between FANCC and STMN1 and CDK1 would impinge on S38-mediated phosphorylation of STMN1 and reduced mitotic kinases would affect subsequent STMN1 phosphorylation resulting in abnormal spindle sizes and centrosome numbers.

Several kinases, including CDK1, mediate STMN1 phosphorylation during mitosis [22, 23]. Interestingly, CDK1 has been shown to interact with full-length FANCC but not a C-terminal region of FANCC with the disease-causing L554P mutation [21]. In the present study, we found that mutations in FANCC including the L554P mutation also affect its interaction with STMN1. Thus, we speculate that FANCC forms a complex with CDK1 and STMN1, promoting CDK1-mediated phosphorylation of STMN1 on serine 38, while subsequent STMN1 phosphorylation events including S16-mediated phosphorylation would be dependent on a functional FA pathway (Fig 10). Consequently, mutations in FANCC would impinge on CDK1-mediated phosphorylation of STMN1 by lack of interaction between its two partners, which would in turn impinge on subsequent phosphorylation events. Another possibility is that reduced expression of kinases found in FA-deficient cells including patient’s bone marrow cells would negatively impact STMN1 phosphorylation during mitosis. More specifically, reduced expression of Aurora B in FA deficient cells would impact STMN1 phosphorylation on serine 16. As a result, this would lead to an increase in STMN1 activity, which would interfere with the spindle formation during mitosis and result in mitotic spindle abnormalities as those observed in FA cells. Because increased STMN1 activity or high expression of STMN1 is frequently associated with cancers, we propose that impaired phosphorylation of STMN1, which is associated with increased activity combined with DNA repair defects in FA cells, accounts for elevated cancer susceptibility in FA patients. In addition, STMN1 overexpression has been associated with poor survival, cancer progression and treatment resistance in several types of cancers including head and neck, ovarian, breast and cervical carcinomas [49]. Therefore, anti-STMN1 therapy in combination to chemotherapeutic drugs has been proposed as a new therapeutic approach to cancer treatment [58–60]. Combining anti-STMN1 to chemotherapeutic drugs has been proven useful in blocking cancer growth in xenograft models [59]. In view of FA patient’s hypersensitivity to chemotherapeutic agents and the occurrence of cancer types often associated with high STMN1 activity, anti-STMN1 therapy may provide an alternative cancer treatment option for FA patients.

## Supporting Information

S1 Fig STMN1 phosphorylation depends on a functional FA pathway. FA-mutant PD220 and PD20 and corrected PD220/A and PD20/D2 cells were immunostained with antibodies against total STMN1 (A and D), STMN1-phospho-Serine 16 (in B and E), or STMN1-phospho-serine 38 (in C and F) along with γ-tubulin during mitosis and visualized using a Carl Zeiss Axio Imager M2 at 100X magnification. White line indicates scale of 1 μm.
(EPS)

S1 Video FANCC localizes with γ-tubulin at centrosomes. Confocal microscopic visualization and 3D reconstruction of 293T cells labeled with anti-FANCC and anti γ-tubulin antibodies using a Olympus IX81-ZDC fluorescent microscope equipped with a Spinning Disc confocal system and the Metamorph NX software.
(MOV)

S2 Video FANCC localizes with Centrin at centrosomes. Confocal microscopic visualization and 3D reconstruction of 293T cells labeled with anti-FANCC and anti-Centrin-2 antibodies using a Olympus IX81-ZDC fluorescent microscope equipped with a Spinning Disc confocal system and the Metamorph NX software.
(MOV)
